# Challenging a Global Land Surface Model in a Local Socio-Environmental System

**DOI:** 10.3390/land9100398

**Published:** 2020-10-21

**Authors:** Kyla M. Dahlin, Donald Akanga, Danica L. Lombardozzi, David E. Reed, Gabriela Shirkey, Cheyenne Lei, Michael Abraha, Jiquan Chen

**Affiliations:** 1 Department of Geography, Environment, and Spatial Sciences, Michigan State University (MSU), East Lansing, MI 48824, USA; 2 National Center for Atmospheric Research, Boulder, CO 80305, USA; 3 MSU Center for Global Change and Earth Observation, East Lansing, MI 48824, USA; 4 Environmental Science, University of Science and Arts of Oklahoma, Chickasha, OK 73018, USA

**Keywords:** Community Land Model, carbon cycle, landscape ecology, model benchmarking

## Abstract

Land surface models (LSMs) predict how terrestrial fluxes of carbon, water, and energy change with abiotic drivers to inform the other components of Earth system models. Here, we focus on a single human-dominated watershed in southwestern Michigan, USA. We compare multiple processes in a commonly used LSM, the Community Land Model (CLM), to observational data at the single grid cell scale. For model inputs, we show correlations (Pearson’s R) ranging from 0.46 to 0.81 for annual temperature and precipitation, but a substantial mismatch between land cover distributions and their changes over time, with CLM correctly representing total agricultural area, but assuming large areas of natural grasslands where forests grow in reality. For CLM processes (outputs), seasonal changes in leaf area index (LAI; phenology) do not track satellite estimates well, and peak LAI in CLM is nearly double the satellite record (5.1 versus 2.8). Estimates of greenness and productivity, however, are more similar between CLM and observations. Summer soil moisture tracks in timing but not magnitude. Land surface reflectance (albedo) shows significant positive correlations in the winter, but not in the summer. Looking forward, key areas for model improvement include land cover distribution estimates, phenology algorithms, summertime radiative transfer modelling, and plant stress responses.

## 1. Introduction

Across much of the world, human impacts are an important component of the land surface [1]. Growth and loss of urban populations [2], changes in certain land areas and habitability [3], and changing agricultural and forestry practices [4,5] have all changed the ways in which energy and matter move through and among landscapes. In an Earth system modeling context, human impacts are mostly represented as changes in the footprint of urban areas and the parameters of those urban areas, changes in the extent and types of agricultural practices, changes in nitrogen deposition, and changes in global atmospheric CO_2_ concentration [6]. These changes over time can in turn impact water movement through a landscape [7], albedo and heat distribution [8], and the carbon cycle through agricultural practices (e.g., tilling, irrigation, fertilization; [4]), nitrogen fertilization [9], and land conversion [10]. While much of the focus in the Earth system modeling community continues to be on ‘natural’ systems (e.g., [11]), more emphasis on human-dominated landscapes would both improve the performance of models, as compared to observational benchmarks, and enhance the relevance of these models to policy makers (e.g., [12]).

Global land surface models (LSMs) are process-based models that operate at relatively coarse spatial scales (typically around 1°) and aim to represent ecosystem processes to inform climate and ocean models. LSMs can be coupled to climate, sea ice, and ocean models to create Earth system models (ESMs), which represent all of the Earth’s major biophysical processes using only a few external inputs—typically incoming solar radiation, atmospheric CO_2_ and land use/land cover changes [13]. ESMs serve as tools to test hypotheses about how the world works and how it may respond to future conditions [14,15]. LSMs generally include energy, carbon, water, and sometimes nitrogen budgets. They track how energy and matter flow through ecosystems on a sub-daily time step, often 30 min, to match the temporal scale of most atmospheric models. While these models vary widely in their complexity and the processes they represent [16], they typically include a few to many plant functional types (PFTs), plant physiology and phenology, below ground processes, and some human impacts (e.g., land use change, agriculture, and/or urban areas). The purpose of these models is to provide information to the atmospheric model for coupling carbon uptake or release, surface roughness and albedo, latent and sensible heat, and water vapor [17]. Even when LSMs are run uncoupled from the atmosphere and oceans, with standardized meteorological forcings, they predict substantively different current and possible future states of the world [18]. Much work has been done comparing LSM predictions to satellite-derived measurements or observationally derived data syntheses (“model benchmarking”), which has revealed areas and processes where models perform relatively well, and/or places where improvements could be made [19–21].

Scientific efforts in improving LSMs are often, however, confined to a single biome or process. For example, LSM benchmarking studies may focus on temperate broadleaf phenology [22], dryland phenology [23], large river hydrology [24], groundwater storage [25], and more. Yet, studies of ecosystems and landscapes often take a more synoptic approach, considering all or many of the flows of materials and energy that enter and leave a geographic area [26]. Benefits of this broader landscape approach are that it allows a deeper understanding of a given ecosystem and the connections between processes.

The overarching objective of this study is to understand how well a commonly used LSM represents multiple connected processes at the local scale. LSMs and ESMs are intended to make global-scale predictions; however, they are often used in decision-making for individual municipalities [27,28]. To assess whether LSMs should be used at the local scale, and how they could be improved, we focus on one watershed in southwestern Michigan (USA), learning about the different components of the model in a single geographic location. This region is particularly interesting because it is part of a large ecotone between the U.S. corn belt (previously the prairie peninsula [29]) and northeastern mixed and deciduous forests. We ask (1) how well does the LSM represent processes in a dynamic temperate watershed, organizing our benchmarks to include model inputs (climate, land cover), direct model outputs (leaf area, productivity), and aggregate model outputs (soil moisture, reflectance)? (2) What can we learn from differences between measurements and the model inputs and predictions? (3) How can this inform future model development and land use planning efforts? By taking this unique approach of assessing multiple processes in one location, we draw new insights into model performance, its relevance for planning efforts, and ecological uncertainties within and beyond LSMs.

## 2. Materials and Methods

### 2.1. Location

**Figure 1.**
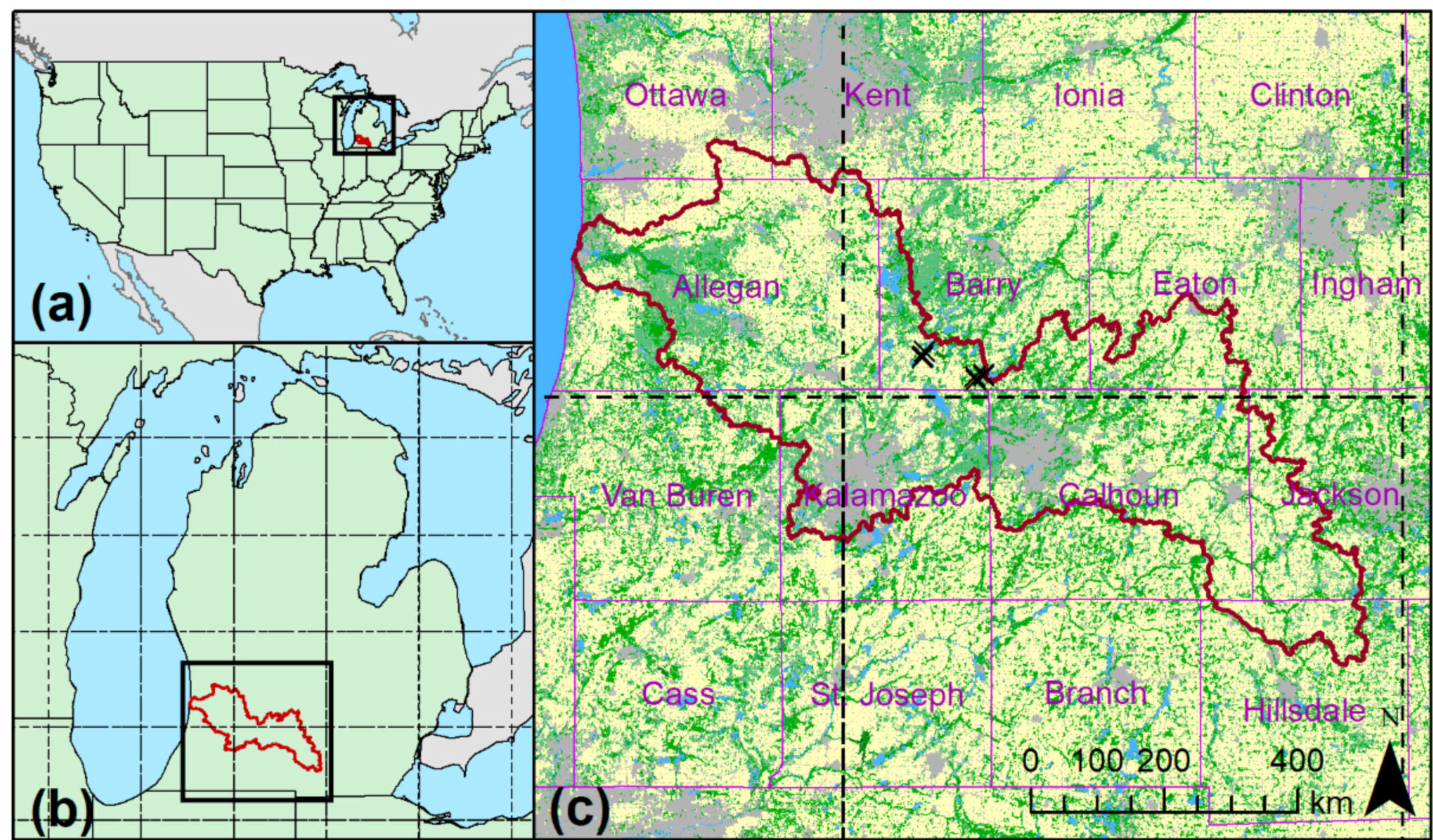
Southwestern Michigan study location—Kalamazoo watershed (KW) outline in red in all panels. (**a**) Map of the contiguous United States showing location of lower Michigan, with black box indicating the boundary of the map in B. (**b**) Map of lower Michigan with boundary for map A shown in the black box. Dashed lines indicate boundaries of Community Land Model (CLM) grid cells in B and C. (**c**) Local map of the KW with counties in purple with a simplified National Land Cover Database (NLCD) 2011 land cover map as background: gray is developed, yellow is cultivated, light green is forest, darker green is wetland, blue is water. Flux tower locations in southern Barry County are marked with a black “x”.

We delineated the Kalamazoo River watershed using USGS Hydrologic Units level 4 ([30]; HUC #04050003). The landscape is underlain by thick glacial deposits topped with prime agricultural soils [31]. Recent average annual precipitation is 790 mm and air temperatures range from −27.8 °C to 40 °C with an annual average air temperature of 9.4 °C [32]. It includes portions of 10 counties (Allegan, Ottawa, Van Buren, Kent, Barry, Kalamazoo, Calhoun, Eaton, Jackson, and Hillsdale) within its 5261-km^2^ extent (Figure 1).

The first people in Michigan arrived shortly after its deglaciation, at least 10,000 years ago [33]. Before European settlement, Neshnabé people (often called Potawatomi) occupied the region, and the landscape was a mosaic of deciduous forest, wetlands, tallgrass prairies, savannas, and oak openings [34]. Today, the two major cities in the watershed are Kalamazoo (population ~76,000; [35]) and Battle Creek (population ~52,000; [35]). Contemporary land cover is representative of the surrounding area of southern and central Michigan—a homogeneous mosaic of agriculture, forests, and urban areas.

### 2.2. Model Description

The Community Land Model (CLM; [36]) is the land surface component of the Community Earth System Model (CESM; [37]). In its offline (uncoupled) mode, the CLM takes in information about the weather, atmospheric CO_2_, and land cover change and predicts change in the physical and biogeochemical state of the planet. Each ~1° × 1° grid cell is divided into up to five land units (i.e., vegetated, crops, deep lakes, glaciers, and urban). Within these land units, the model includes 14 different non-crop plant functional types (PFTs), up to 64 crop types, predictive models of phenology for natural vegetation and crops, and variable wetlands (i.e., wetlands are defined by inundation, not as a PFT). Most of the PFTs present in our study follow CLM’s seasonal-deciduous phenology algorithm, whereby growth onset is triggered by a combination of growing-degree days and offset is triggered by daylength [37]. Seasonal maximum leaf area index (LAI) is determined by the available C and N from the preceding growing season. Here, we used output from CLMv5.0 run from 1700 through 2017 with CRU-JRA version 1 forcing data [38,39] including land use change and changes in [CO_2_]. These model runs were performed as part of the 2018 Global Carbon Budget report focusing on the S3 simulations [38].

Due to the scale mismatch between the watershed and the CLM grid (see Figure 1), we focused primarily on the entire area encompassed by the two CLM grid cells that include the eastern side of the Kalamazoo River watershed. For simplicity, we refer to the actual Kalamazoo watershed (KW; dark red line in Figure 1), while the northeastern CLM grid cell that intersects KW is referred to as CLM-N and the southeastern grid cell is CLM-S. Where available, we compare CLM data from the two grid cells to satellite products at all three spatial extents—KW, CLM-N, and CLM-S. As the western grid cells contain substantial fractions of Lake Michigan, which complicates process summaries in CLM outputs, we excluded them in this analysis.

### 2.3. Benchmarking Data and CLM Comparisons

**Figure 2.**
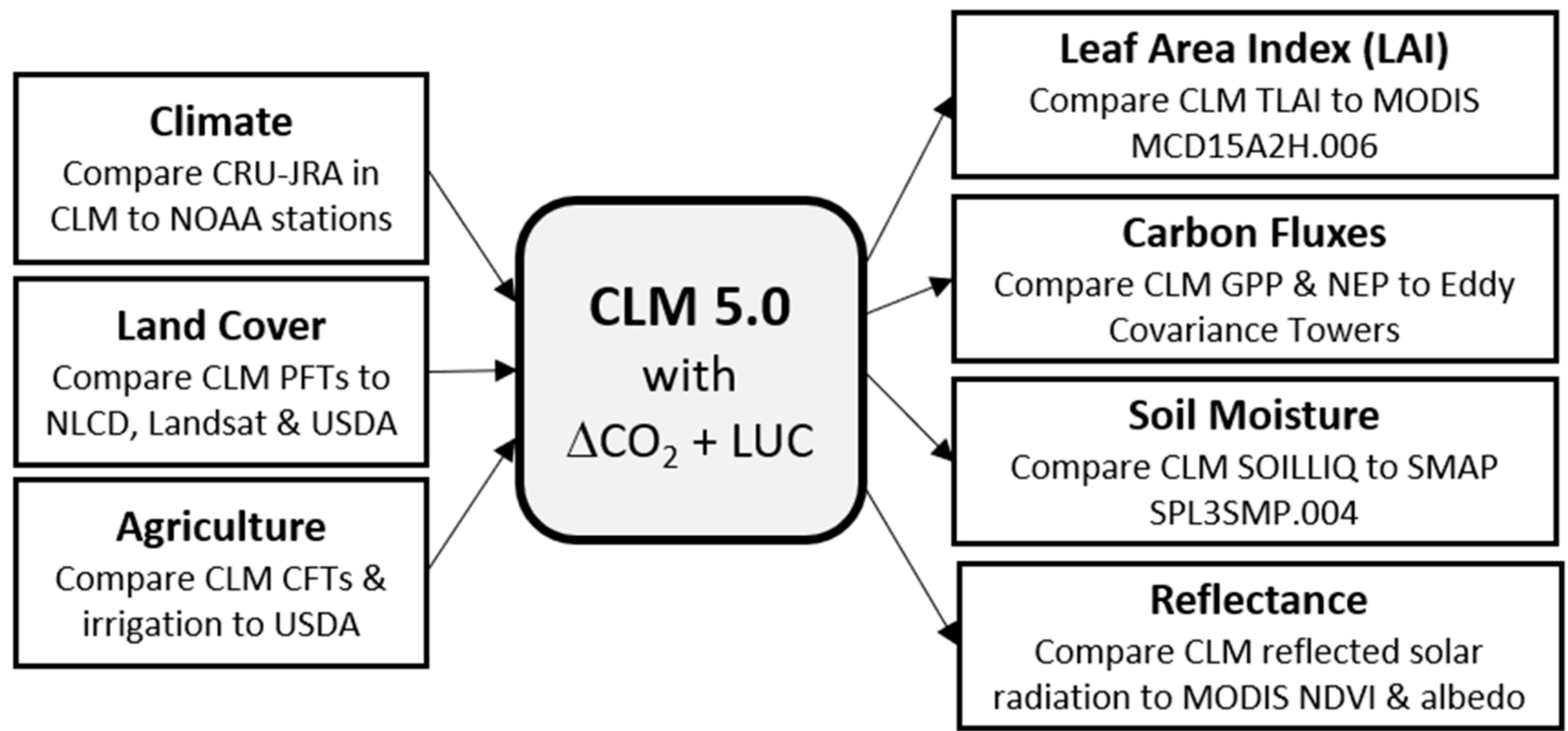
Process diagram of CLM data input (left) and output (right) comparisons made in this study. All abbreviations are defined in the text.

To compare observations to the LSM, including both inputs (e.g., climate, land cover change) and outputs (e.g., gross primary production (GPP); Figure 2), we gathered data from different sources which were available at different spatial and temporal resolutions.

#### 2.3.1. Climate

Meteorological data for 1900 to 2017 were obtained from weather stations in six counties that substantially overlap the KW (Allegan, Barry, Calhoun, Eaton, Jackson, and Kalamazoo, see Figure 1) via the US National Oceanic and Atmospheric Administration’s National Centers for Environmental Information (NCEI; https://www.ncdc.noaa.gov) accessed via the “rnoaa” R package [40]. We compared temperature and precipitation over the available data series (1900–2017) and over the recent past. NOAA data with any quality flags indicating issues with the data were removed from further analysis.

NOAA temperatures are provided as daily minimum and maximum values for each station, which we compared to CLM monthly averages of daily minimums and maximums. To compare these values, we first aggregated the NOAA values to daily minimum and maximum values by county, calculating the mean of multiple observations if two or more stations in a county reported values for the same day. We then aggregated these daily values to monthly means of daily minimum and maximums for each county then averaged among counties for quantitative comparisons.

The CRU-JRA forcing data set used with CLM is a combination of CRU monthly data [41] with JRA-55 6-hourly forcing [42], which originates as a 0.5° × 0.5° grid. The data set includes precipitation, temperature, and incoming shortwave radiation, among other variables. These values are spread or interpolated to match the 30-min time step of CLM, then average monthly values are written out as CLM outputs when a CLM run is executed.

Station-based precipitation (mm) was calculated as annual and monthly sums from reported daily values. For each county, daily averages were calculated if multiple station readings were available for a given day, then daily values were summed to monthly and annual. Monthly and annual values were then averaged across the six counties. CLM precipitation was calculated by adding the RAIN and SNOW variables (both in mm s^−1^) then scaling to monthly sums by multiplying by the number of seconds in each month.

#### 2.3.2. Land Cover and Plant Functional Types

Overall land cover was assessed using three sources. We completed our own land cover classification of KW using Landsat imagery from 1976, 1981, 1986, 1991, 1996, 2001, 2006, 2011, and 2015 [43]. In addition, we extracted land cover information from the USGS National Land Cover Database (NLCD; [44]) for 2001, 2006, 2011, and 2016. To understand the land cover prior to European settlement, we relied on a pre-European settlement map of vegetation developed specifically for Michigan [45], hereafter referred to as the 1800 map. This map was developed based on the General Land Office surveyor notes collected between 1816 and 1856. As this is a Michigan specific data set, it does not include the part of the CLM-S grid cell that falls in Indiana (Figure 1). We aggregated the land cover to a percentage of its total coverage and assumed the northern ~60% of the grid cell is representative of the overall land cover.

Land cover in CLM is described via fractional cover of PFTs. PFT distributions are determined from the Land Use Harmonized version 2 (LUH2) database [46,47]. The relationship between LUH2 and CLM PFTs is described in [48]. LUH2 was developed to merge historical estimates of land cover (from 1500 to 2005) with future predictions of land cover change (2006 to 2100). The distribution of urban environments is described in [49].

These land cover descriptions use different schema; for example, NLCD includes a “mixed forest” class, while in CLM this would be represented as fractions of different forest types. To compare across classification types, we developed a cross-walking procedure to connect the different land cover types (Table S1). Then, to compare CLM to these land cover classifications, we plotted the percent cover of each coarse land cover type (e.g., forest, grass, crop) over time from 1800 to 2017. For the recent past, we also compare CLM to NLCD. The 1800 map cross-walking table, which contains many specific vegetation types, is in Supplemental Table S2.

#### 2.3.3. Crop Cover and Irrigation

The U.S. Department of Agriculture has provided a spatially explicit representation of crop cover for the U.S. via the CropScape webpage [50]. Michigan data are available in a consistent classification since 2008. CropScape is produced using a combination of satellite imagery (mostly Landsat and Sentinel-2) and ancillary information about topography and soils. Accuracy assessment and development information is provided by state for every year (www.nass.usda.gov/Research_and_Science/Cropland/metadata/meta.php). Non-cropland cover in these maps is derived from NLCD. Here, we extracted CropScape information for the KW boundary and the two CLM grid cells for each year. As these data are only available for a short period of time that overlaps CLM (10 years) and there was relatively little change in land cover over that time period, we compared 2016 values between CLM, NLCD, and CropScape.

Total farmland acreage and acreage of irrigated farmland were obtained from the USDA National Agricultural Statistics Service (NASS) for each census of agriculture (i.e., 5 years) since 1950 for all 10 counties in the study area. This data is a complete account of county-level farms and ranches, as well as the demographics of owners and operators, that produce more than USD 1000 in fruit, vegetable or animal products. Irrigation includes all methods, including sprinklers and drip systems. Farmland acreage represents total acreage of agricultural land owned and managed of all farm operators per county [51–64].

#### 2.3.4. Leaf Area Index and Phenology

LAI (m^2^ m^−2^) was assessed by comparing the MODIS LAI product (MCD15A2H.006; [65]) which is at 500 m spatial resolution and 8-day temporal resolution. GeoTIFFs of the KW as well as the CLM-N and CLM-S grid cells were downloaded via the NASA/USGS AppEEARS system (https://lpdaac.usgs.gov/tools/data_access/appeears) on 19 October 2018. Data were downloaded for 1 January 2003, through 31 December 2017. LAI values were then averaged within each area to provide a single LAI value for each time period.

#### 2.3.5. Carbon Fluxes

Carbon cycling was measured using seven eddy covariance (EC) towers located within the KW (Figure 1). The towers were located in a series of agricultural fields and restored prairies (see [66] for site information). Since these towers are all located in agricultural or grassland sites, they do not represent the landscape as a whole, however, they can still provide some information about the magnitude and timing of C fluxes in this region.

Each tower was equipped with an infrared gas analyzer (LI-7500, LI-COR Biosciences, Lincoln, NE, USA) and a 3D sonic anemometer (CSAT3, Campbell Scientific Inc., Logan, UT, USA), with sensor heights 1.5–2.0 m above canopy heights [67]. Fluxes were processed following Ameriflux guidelines, with 30-min average fluxes (i.e., net ecosystem production, NEP) computed with EdiRe [68] and then gap filled and partitioned into gross primary production (GPP) and ecosystem respiration using REddyProc [69]. Flux data was u* filtered [70], with an average u* threshold of 0.11 m s^−1^, removing 25.3% of the data.

#### 2.3.6. Soil Moisture

Soil moisture fraction (m^3^ m^−3^) was measured using NASA’s soil moisture active passive (SMAP) radiometer global product version 4 (SPL3SMP.004; [71] https://nsidc.org/data/SPL3SMP/versions/4) at 36 km spatial resolution and daily temporal resolution. SMAP was launched in 2015. Similar to LAI, GeoTIFFs of the KW as well as CLM grid cells were downloaded via the NASA/USGS AppEEARS system on 19 October 2018. Data were downloaded for 31 March 2015(earliest available data), through 31 December 2017. Data was filtered to remove records with quality flags. AM and PM measurements were then averaged to produce a daily measurement. If only one measurement was reported on a given day, we used that measurement alone. Initial comparisons showed that the intra-day differences were minor relative to seasonal variation.

To match the SMAP data, CLM values were aggregated for the first two soil layers (0–0.06 m). Only three growing seasons were available from SMAP, so unlike the other data products, SMAP maximum, mean, and seasonal patterns were compared qualitatively to CLM, but not quantitatively.

#### 2.3.7. Reflectance

The interaction between incoming solar radiation and the land surface is both important as a basic remote sensing tool and from an ESM perspective as albedo is a critical determinant of land surface temperature. Albedo is also an integrative model component as it is determined by several factors, including land cover, snow cover, management practices, and LAI. Here, we consider reflectance from two remotely sensed MODIS products. We consider NDVI from the 500 m eMODIS temporally smoothed weekly AQUA collection 6, downloaded from the NASA/USGS AppEEARS system (USGS, 2019). We also consider the 500 m daily MODIS albedo products (MCD43A3v006; [72])—black and white sky albedos in both the visible and the near infrared. We processed the albedo products and applied mandatory quality flag mask using Google Earth Engine (GEE [73]). In the case of both the MODIS NDVI and albedo products we calculate the spatial means for each time step for the CLM-N, -S, and KW areas. Since the MODIS albedo product is produced at daily temporal resolution, we aggregated these to monthly (averaged, ignoring missing values) to compare to CLM.

In the CLM, light interacts with the land surface through a two stream radiative transfer modelling approach [74]. Incoming solar radiation (W m ^−2^) is provided by the CRU-JRA forcing data set and is partitioned into direct at local noon and diffuse streams in visible and near infrared (NIR) bands. Reflected light is then calculated in CLM for those same streams via radiative transfer. To compare to MODIS NDVI, we calculated reflectance for visible and NIR wavelengths as the ratio of the sum of direct and diffuse reflected radiation to the sum of those incident values. We then calculated NDVI as the difference between the NIR and visible reflectance values divided by their sum. To compare albedo values, we compared CLM NIR and visible, direct and diffuse reflectance to MODIS NIR and visible, black and white sky albedos, respectively [75].

### 2.4. Model Data Comparisons

To compare time series between CLM and the data streams, we calculated annual maximums or absolute maximums, and means and present both Pearson correlation coefficients (*R*) and Spearman rank correlation coefficients (*ρ*) of annual summary values. To qualitatively compare intra-annual variations visually, we temporally aggregated data streams to monthly then plotted monthly time series for five years, when available, centered around the 2012 drought [76]. In addition, to understand how the CLM values related to the spatial variability within the three extents (the two CLM grid cells and the KW), we concatenated average monthly summer values (June, July and August) for each year available for each data type in its original resolution then computed a probability density function (PDF) for each data set. This allowed us to compare the CLM summer values to the range of observed values across the areas. In the case of albedo, we also compared winter values (December, January, and February) to CLM.

The field observations and remotely sensed data sets considered here also contain inaccuracies that could impact model-data comparisons. Some of the data streams considered here are near direct measurements, like the weather station data, NDVI, and albedo. Whereas landcover classification, LAI, productivity, and soil moisture all require some amount of data manipulation and modeling. Here, for simplicity, we do not fully explore these uncertainties, and focus on large differences between model and data estimates. The field and remotely sensed observations should not, however, be viewed as perfect measurements of the values they represent.

## 3. Results

### 3.1. Climate Forcing

**Figure 3.**
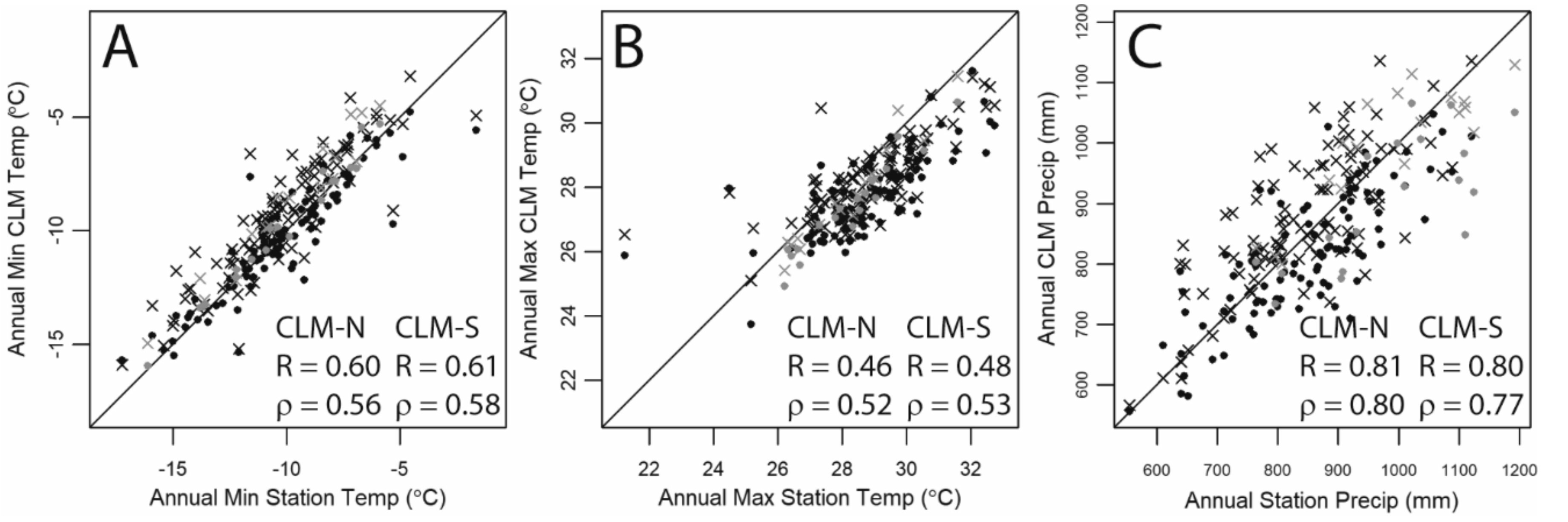
Scatterplots of (**A**) annual minimum of monthly average minimum temperatures; (**B**) annual maximum of monthly average maximum temperatures; and (**C**) total annual precipitation. All correlation values are significant (*p* < 0.01). CLM-N are dots, CLM-S are represented by “x”. Blue dots and x identify years from 2000 to 2017. The solid line is the 1:1 line.

Over the 119 years of weather observations, the mean of minimum monthly temperatures in CLM (CRU-JRA forcing) was 4.6 °C in CLM-N and 5.4 °C in CLM-S, whereas in NOAA data this was 3.4 °C. These minimum temperature means were significantly different (t-tests, *p* < 0.001) over the course of the time series. For monthly maximum temperatures, CLM-N average monthly maximum was 13.1 °C, CLM-S was 13.9 °C, and the NOAA data was 14.6 °C. Here, however, the CLM-S mean was not significantly different from NOAA (t-test, *p* = 0.1). When comparing the time series, the average annual NOAA values were significantly correlated with the CLM values (Figure 3A,B).

For precipitation, over the 119 years of weather observations, total annual precipitation in CLM averaged 830 mm in CLM-N, and 897 mm in CLM-S, whereas the mean annual precipitation in the NOAA data was 867 mm. The means were marginally different between the two CLM grid cells and the NOAA data (t-test, CLM-N *p* = 0.02; CLM-S *p* = 0.05). As with temperature, the annual values were significantly correlated.

**Figure 4.**
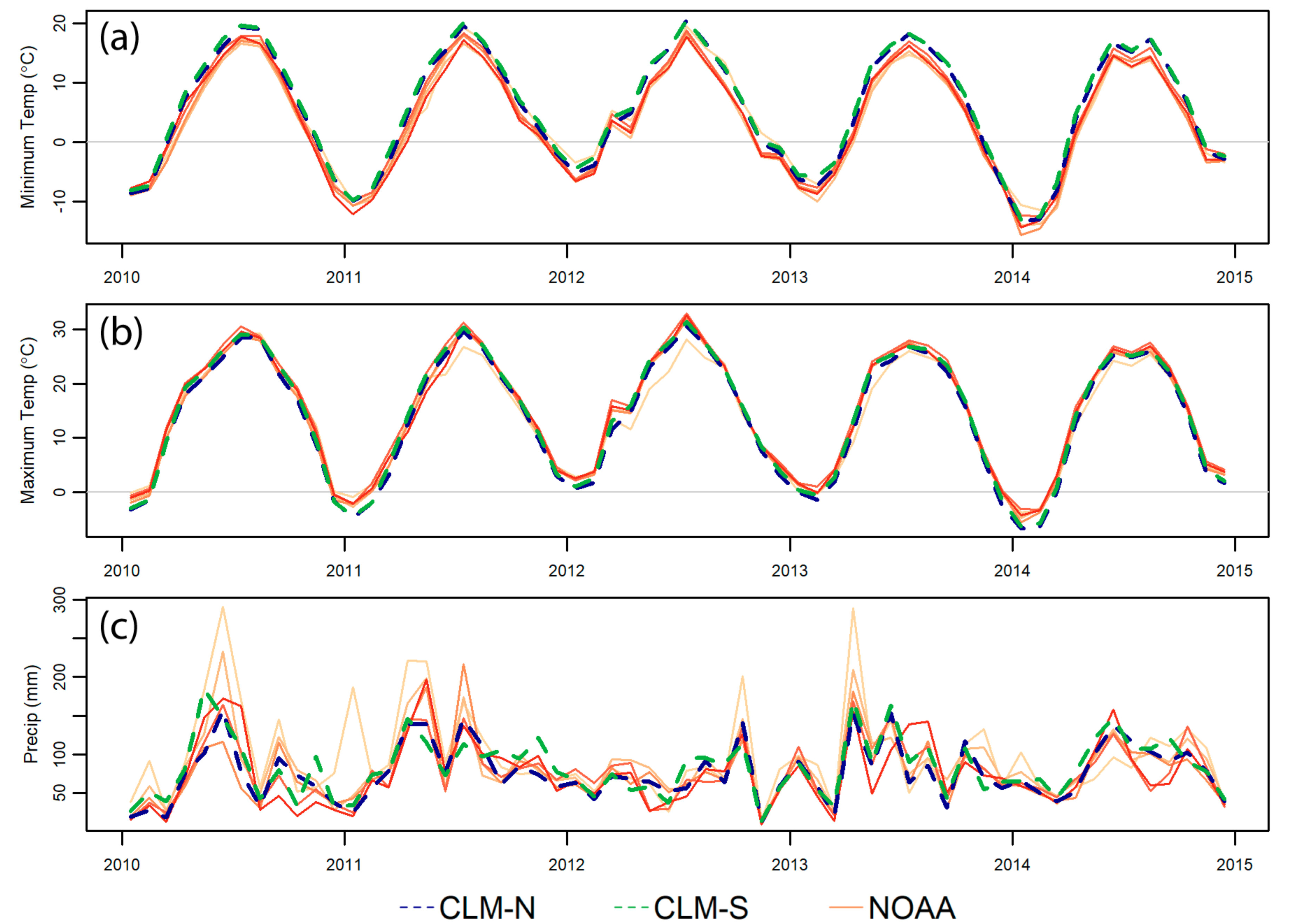
Comparison of average monthly minimum temperature (**a**), monthly maximum temperature (**b**), and total monthly precipitation (**c**) values for the five years surrounding 2012. Station data are warm solid lines—each line represents a different county.

To compare the seasonality of temperature and precipitation we considered the monthly patterns over the 5 years surrounding 2012 (Figure 4). Temperature values track very well (Figure 4a,b); however, it appears that the CRU-JRA climate forcing data consistently underestimates high precipitation months (Figure 4c). The climate forcing data does, however, capture the elevated winter and spring temperatures and lowered precipitation in the 2012 drought.

### 3.2. Land Cover Forcing

**Figure 5.**
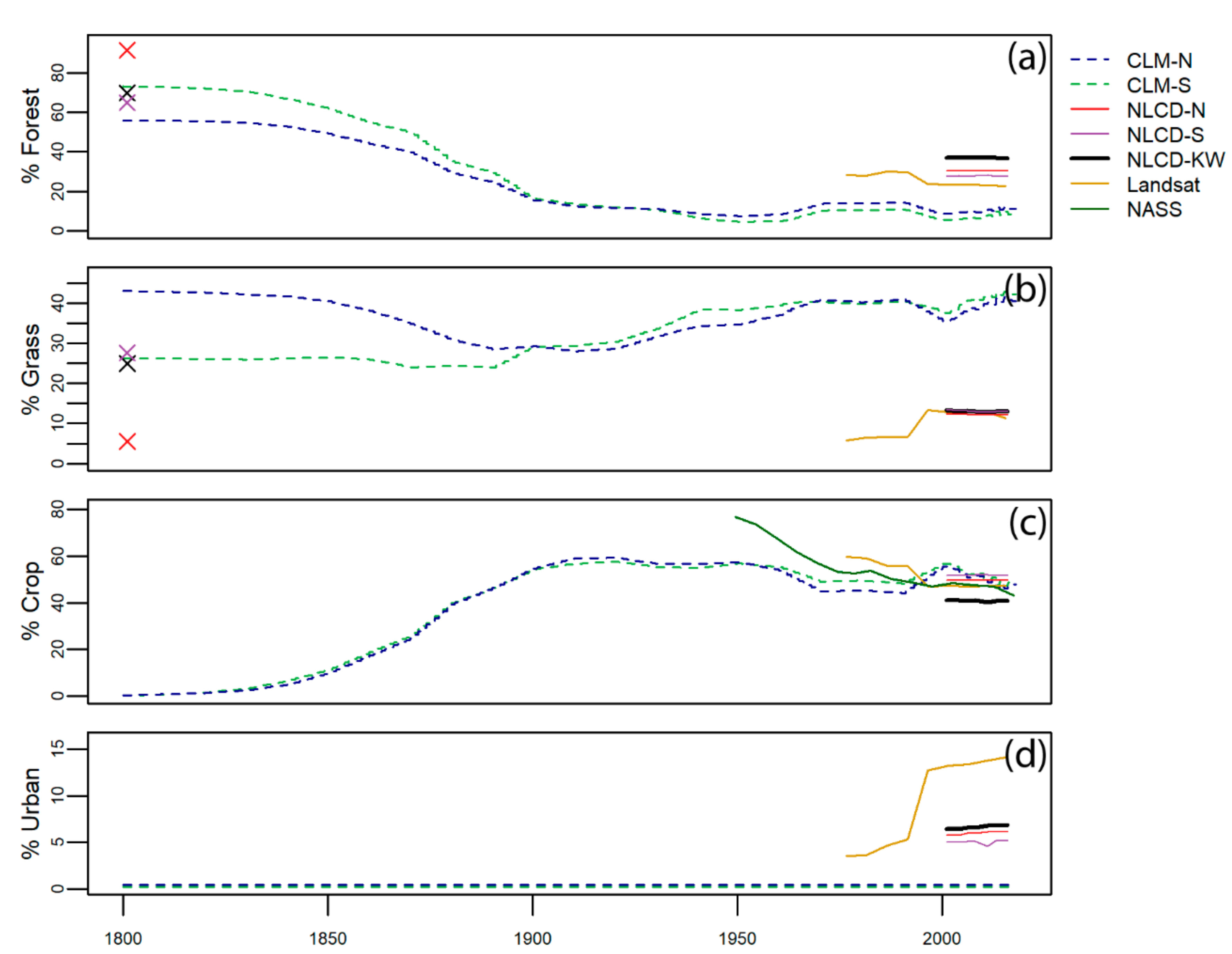
Long term comparison of landcover in CLM to available data (note varying y-axes). The 1800 map vegetation data for forest and grass are represented by an “x” at 1800 with colors corresponding to the two CLM grid cells as above and black for the KW. Plots show forest (**a**); grasslands (**b**); croplands (**c**); and urban areas (**d**). NLCD is the Landsat derived National Landcover Database. “Landsat” refers to the classification described in Chen et al. (2019). NASS = USDA National Agricultural Statistics Service.

To look at long term changes in land cover we used the 1800 map. We compare these single points in time to CLM for the same time period (Figure 5). CLM does not include any unvegetated land or deep lakes in these grid cells and are therefore not presented, though they made up 3–7% of the land cover in the 1800 map. This comparison shows that generally the CLM captures the coarse vegetation patterns shown in the 1800 map, especially in CLM-S. However, in CLM-N there was considerably less grass and more forest in the 1800 map than is represented in CLM.

Our project-specific Landsat-based classification starting in 1976 for the KW shows that, generally, the CLM gets the average amount of crop area correct: 48% and 51% in CLM-N and S, respectively, versus 52% in the classification, and similar in the NLCD (Figure 5C). The NASS % crop cover data also corresponds to the other data sets for the recent past. In the middle of the 20th century; however, according to NASS, aggregated for the same six counties as in the weather data, this region was nearly 77% cropland, dropping steadily to 43% in 2017. Forest cover is 26% in the Landsat classification versus 12% and 9% in CLM-N and S. CLM substantially over-represents grasslands, showing very little change in the grass coverage over this >200-year time period (~40% cover over the Landsat time period versus ~12% in the classification and NLCD). Moreover, in our Landsat classification and NLCD, we combined herbaceous wetland, grassland, and (for NLCD) pasture and “urban-open space” classes, so this is a generous estimate of grassland and herbaceous cover for the area. Urban areas are also not well represented in CLM, staying at <1% throughout the entire period. In contrast, our classification shows a rapid increase in urban areas in the watershed from 1976 to the present. NLCD estimates urban cover at 5–7%. Overall, CLM represents this area as a mixture of croplands and grasslands, with a small amount of forest, whereas in reality, the area is a mixture of croplands and forest, with small amounts of grasslands and urban areas.

To compare more specific land cover types to PFTs, given the minimal change in land cover over the NLCD period we only compared one year—2016 (Table S3). We found that in terms of absolute numbers, broadleaf deciduous forest is the most underrepresented in CLM, while C3 grass is over-represented. By considering the normalized differences as well, we emphasize the relative differences between smaller land cover fractions. Here, we see show that high and medium density urban are very poorly represented, as they are almost non-existent in CLM, but over 0.5% and 1%, respectively, of the land cover in NLCD. Low-density urban areas make up the remainder of urban land mapped in NLCD, but this class is not represented in CLM and, therefore, not shown in Table S3. Similarly, bare ground is not present in CLM but makes up a small fraction of the landscape (>0.1%) in NLCD.

### 3.3. Land Use Forcing

USDA’s relatively new CropScape product, which is only available since 2008 in Michigan, identifies >50 crops grown in the KW and CLM grid cells. Of these, the ones making up >1% of total land cover are corn (16–21%), soybeans (10–18%), alfalfa (3–5%), and winter wheat (2–4%). In contrast, CLM lists three crops grown in the past decade—corn (18–22%), soybeans (18–22%), and spring wheat (5–10%).

**Figure 6.**
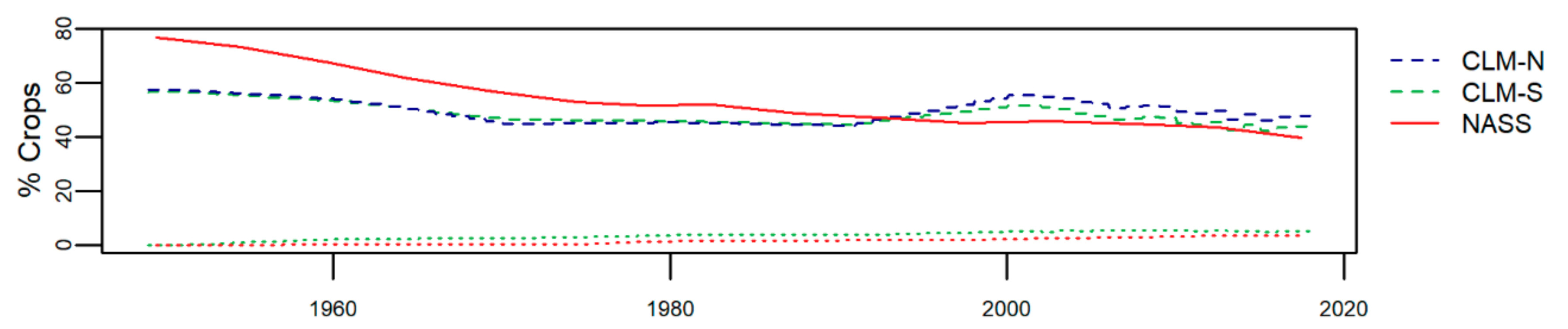
Comparison of crop cover and irrigation over time. Irrigated area is plotted with the same colors as non-irrigated, but with dotted lines. There is no irrigated land in CLM-N.

Irrigation information from USDA NASS and CLM tell relatively similar stories (Figure 6). There is no irrigation in CLM-N and only a minor fraction in CLM-S. In the NASS data, where 1949 is the first available year of data, a small amount of irrigation was documented (<0.1% total land area). Over the subsequent decades this amount has increased slightly to 3.6% in 2017. In CLM, irrigation in CLM-S begins in 1951 (0.22% of total land area) and increases to 5.2% in 2017.

### 3.4. Model Output Time Series

**Figure 7.**
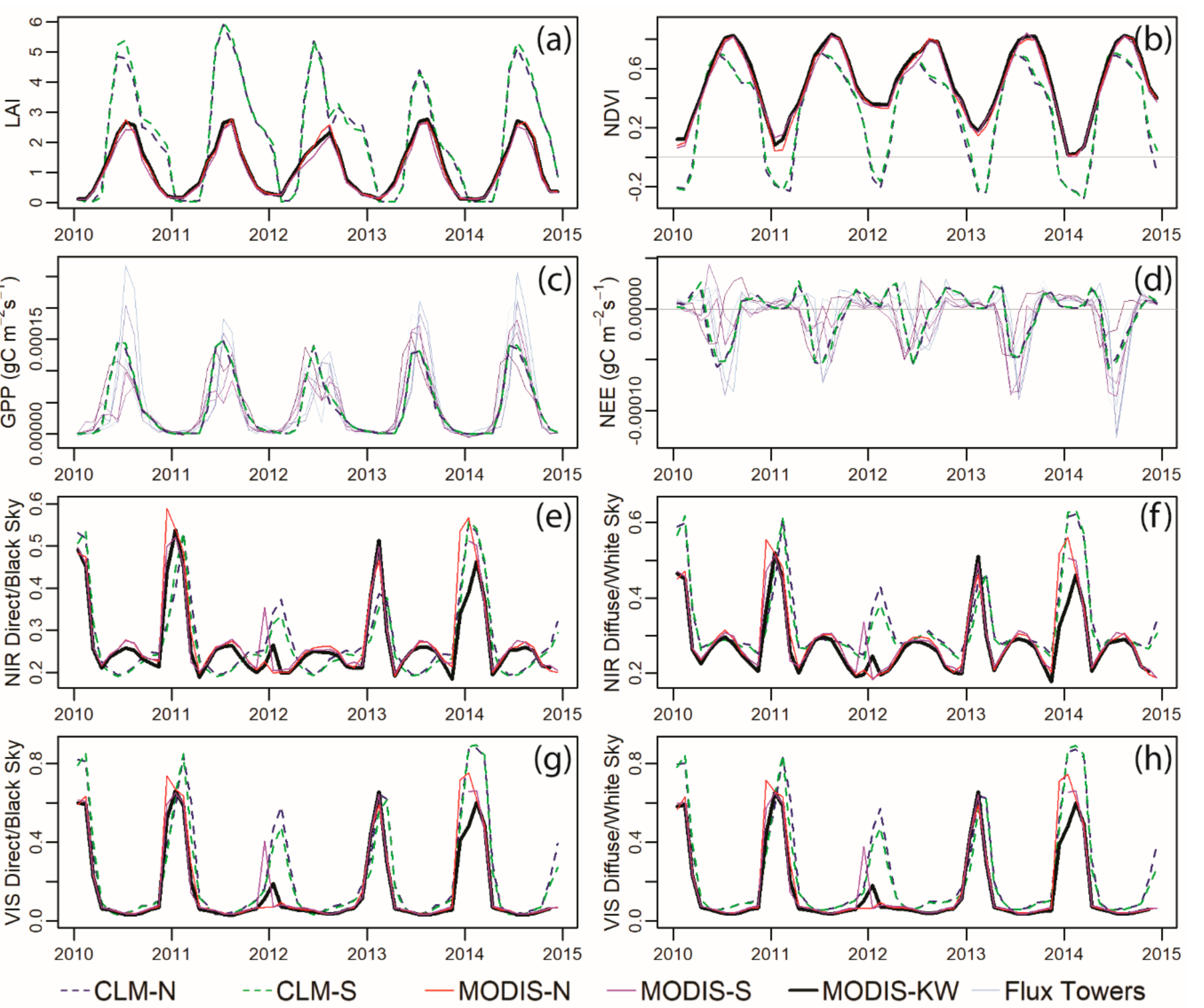
Model output time series compared to observations or satellite products for the five years surrounding 2012 (drought year). (**a**) Leaf area index (LAI); (**b**) NDVI; (**c**) gross primary productivity (GPP); (**d**) net ecosystem exchange (NEE); (**e**) near infrared (NIR) direct or black sky albedo; (**f**) NIR diffuse or white sky albedo; (**g**) visible (VIS) direct or black sky albedo; (**h**) VIS diffuse or white sky albedo.

We compared model outputs—LAI, NDVI, productivity, albedo, and soil moisture—between CLM and satellite or ground observations over the available time series for each data product. In all cases the different spatial extents (the two CLM grid cells and the three areas—CLM-N, -S, and KW) showed similar patterns so values are averaged among these products for quantitative comparisons, but plotted separately (e.g., Figure 7).

To assess how well CLM predicted land surface phenology (LAI) and its magnitude we compared MODIS combined LAI (MCD15A2H.006) for 2003 to 2017. The annual mean values for the two data streams were different—mean annual MODIS LAI was 1.1, while mean annual CLM LAI was 2.1, and the mean values over the 15-year period were significantly different (t-test *p* < 0.001). Maximum LAI values from MODIS (2.8) were also significantly different from the annual maximum values in CLM (5.1) (t-test *p* < 0.001). Correlations between the data streams annual mean and maximum values were insignificant (*p* > 0.1) for both Pearson’s R and Spearman’s ρ. As the CLM data for these simulations was available in monthly time steps and MODIS is in 8-day time steps, we did not quantitatively assess phenological timing (e.g., green up, peak greenness, etc.); however, from Figure 7A, it appears that the timing of peak greenness matches relatively closely, while green up and senescence timing are misaligned. Senescence in CLM appears to be drawn out, sometimes into January, which does not align well with MODIS LAI estimates or our personal observations in Michigan.

We compare carbon flux information which was available from seven EC towers operating in agriculture and restored prairie systems in the KW from 2010 through 2017. Overall, the annual maximum NEP uptake and GPP values from the towers (annual |maximums| of monthly means = 9.80 × 10^−5^ and 2.18 × 10^−4^ gC m^−2^ s^−1^, respectively) were significantly different from the annual maximum values in CLM (annual |max.| of monthly means = 5.51 × 10^−5^ and 1.42 × 10^−4^ gC m^−2^ s^−1^) (two sided t-tests, *p* < 0.01). Correlations between the data streams for annual mean and max GPP and NPP as both Pearson’s R and Spearman’s ρ were all non-significant (*p* > 0.1). We note that 8 years is a short period of time to detect correlations; however, as displayed in Figure 7C,D, CLM shows very limited interannual variability in carbon fluxes, remaining at values more similar to the 2012 drought year, while the EC towers show substantial interannual variability as well as spatial differences.

The maximum NDVI values for the two data streams were significantly different (t-test *p* < 0.001), with the MODIS NDVI average as 0.83, while the CLM average was 0.69. Over the 15-year time series the maximum values were not significantly correlated (Pearson’s R and Spearman’s ρ both *p* > 0.1). Similarly, the mean values for the two data streams were significantly different; CLM mean NDVI was 0.30, while MODIS mean NDVI was 0.50 (t-test *p* < 0.001). The mean NDVI values were marginally correlated between the two data sets, with a Pearson’s R of 0.60 (*p* = 0.019) and a Spearman’s ρ of 0.50 (*p* = 0.058). The eMODIS composite selection algorithm selects the brightest reflectance values and removes snowy pixels unless all the values are snowy. Therefore, we would expect the MODIS data to have higher winter NDVI values than from direct, unfiltered observations. As such, we do not quantitatively compare minimum NDVI values between CLM and MODIS. However, the CLM minimum values (all below zero) are at the lower limit of what would be expected for a wintertime NDVI [77]; in order to achieve these low values in real life, the entire grid cell would need to be completely snow covered for an entire month, which is unrealistic in southwestern Michigan [78].

To understand the separate components of reflectance we compared albedo values—white sky (diffuse) and black sky (direct) in the visible and NIR wavelength ranges. These patterns are dominated by different processes seasonally, with green vegetation in the summer and snow in the winter. As such, we divided our analysis into two five-month segments that we refer to as summer and winter and compare mean and maximum values for the summer (June–October) and winter (December–April). For the summer values, while CLM means and maximums matched the orders of magnitude of the MODIS albedo products, in all cases the means were significantly different (Table S4). In considering the time series, none was significantly positively correlated, and in some cases the mean and/or maximum values were negatively correlated between CLM and MODIS. For winter values, the mean and maximum values were still significantly different between the two data streams, but all the time series were positively correlated (Table S4).

A visual comparison of the time series (Figure 7E–H) shows reasonable timing and magnitude for winter albedo values (large peaks), though CLM values are substantially brighter than MODIS. In contrast, summer patterns are much more variable, especially in the NIR. In all four cases, the 2012 drought is clearly captured in the winter, presumably because the drought resulted in lower than normal snow cover, as suggested by the higher than average minimum temperatures in that year (Figure 4A).

**Figure 8.**
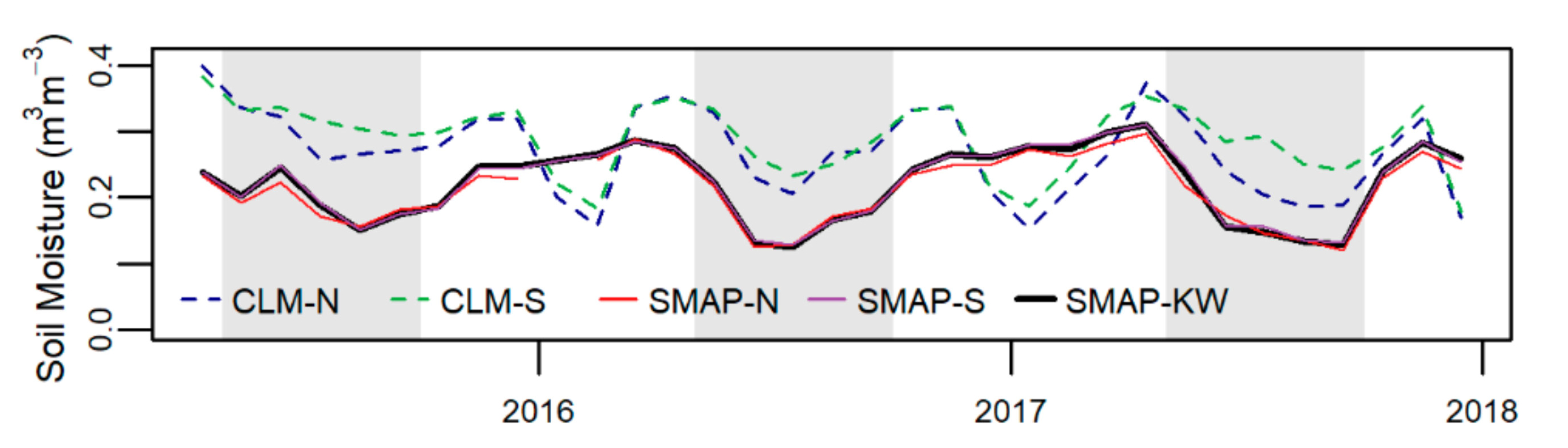
Comparing soil moisture from the SMAP satellite, which measures the top 5 cm of soil, to the top two layers of CLM soil (6 cm). SMAP launched in early 2015, so data is only available from March 2015, onward. Gray boxes highlight the approximate growing season—May to September.

To understand how climate and weather might influence phenology and productivity in this watershed we compared CLM-estimated soil moisture to that measured by the SMAP satellite. As the overlap between the two data streams is only three growing seasons, we did not quantitatively compare these patterns. However, as shown in Figure 8, while the seasonal patterns of CLM and SMAP are similar, there are large differences in the magnitude of soil moisture during the growing season (here defined as May to September). Across the three growing seasons, SMAP mean soil moisture was 0.17 m^3^m^−3^, whereas CLM was 0.28 m^3^ m^−3^. For comparison, a recent study of southern Michigan forest soil moisture found values ranging from approximately 0.22 to 0.24 m^3^m^−3^ for healthy forest soils [79]. The wintertime soil moisture estimates between the two data streams do not match. However, due to the spatiotemporal heterogeneity of freeze thaw cycles in this region, it is likely that both the satellite and model estimates are inaccurate and so we do not consider them further.

### 3.5. Spatial Variation

**Figure 9.**
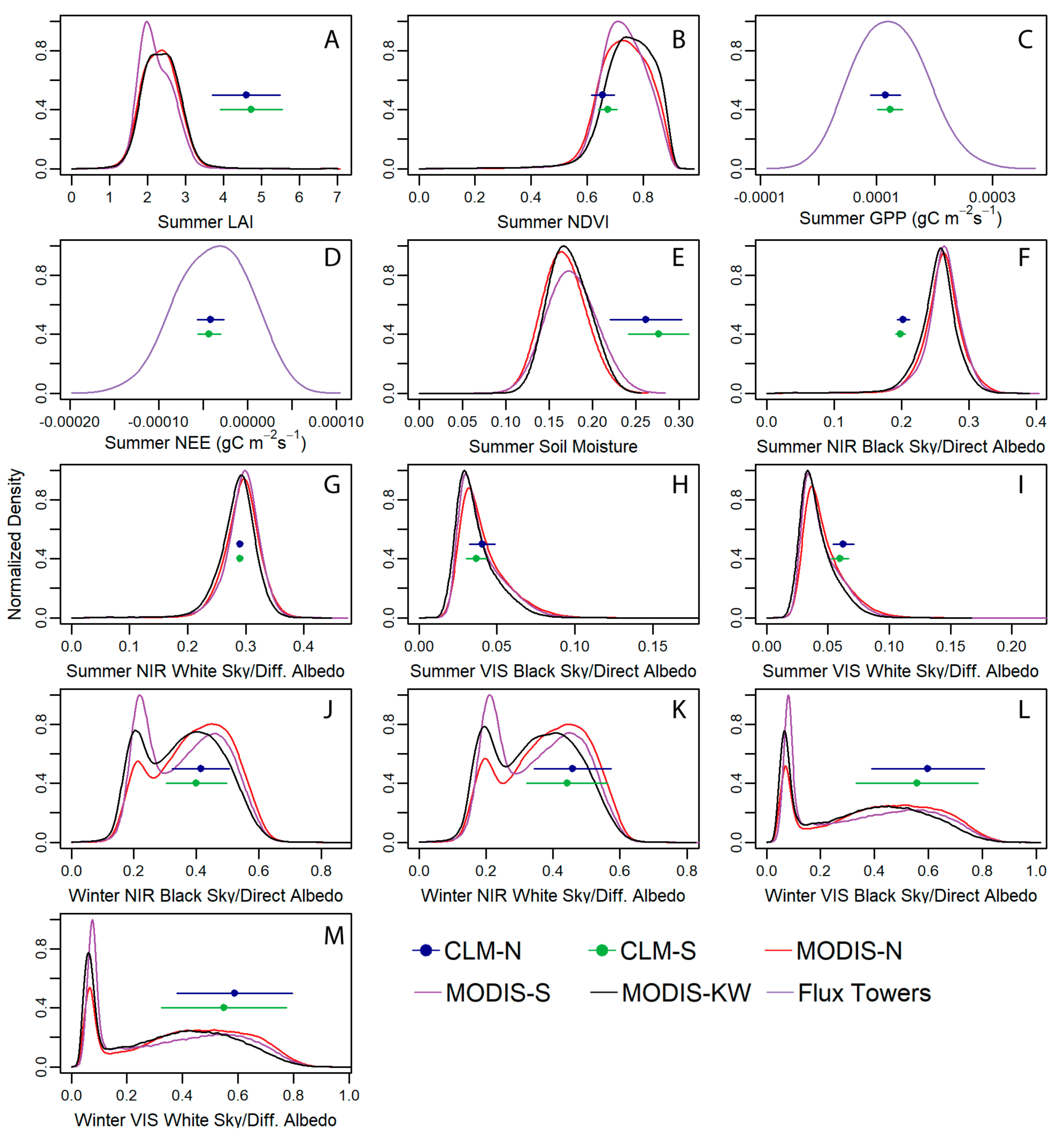
Normalized probability density functions for summer (June, July, August, **A**–**I**) or winter (December, January, February, **J**–**M**) observations including spatial heterogeneity within the grid cell and watershed boundaries. CLM values are placed arbitrarily on the y-axis for visibility.

As the CLM grid cells and KW are averaging across a large, heterogeneous area, we also compared CLM outputs to the range of values present within the areas for the different data streams. To do this we aggregated monthly data across all years of available data from the summer months (and winter for albedo) and calculated PDFs for each distribution. We then plot these in comparison to CLM values across the same three months (June, July, and August, plus December, January, and February for albedo) for 2001 to 2017. We show that for some values, like LAI (Figure 9A), soil moisture (Figure 9E), and summer NIR direct albedo (Figure 9F) CLM values fall outside most observed values. However, for the other measures considered, productivity and the other forms of albedo, CLM values fall within the observed range. Interestingly, for winter albedo the MODIS values show a bimodal distribution, likely due to the presence of forest cover, with low albedos, versus snow covered fields, with higher albedo values (Figure 9J–M). The CLM values align well with the brighter peaks in winter albedo.

## 4. Discussion

### 4.1. Model/Data Inputs (Dis)Agreement

Overall, we found that the climate data used in these simulations (CRU-JRA; [38]) represented the observed climate well on an annual time step, with strong correlations between NOAA station data and the climate inputs to CLM. When considering recent monthly data, precipitation patterns appear dampened relative to the observations, where months are substantially less wet in CLM than in the station data. It is not surprising that an interpolated data set would smooth over extreme weather events [80] and consistently underestimate rainfall in the wettest months. However, especially during the growing season, this difference should have cascading impacts on plant productivity.

We show that the CLM PFT distribution, adapted from LUH2, does a good job of generally representing the crop fraction in this region in recent decades (~40%), but underestimates the amount of forests and urban areas, assuming instead that a large fraction of this landscape is grass. In comparing the pre-European settlement land cover estimates to CLM, it is surprising that CLM represents more grass in CLM-N and slightly more forest in CLM-S. The likely impacts of this difference between CLM land cover and reality matter to both the carbon and energy cycles. A grassy environment stores less aboveground biomass than forests and, in CLM, has a shallower rooting depth [74], which effects water access and therefore transpiration. In the winter, a grassy environment would also be a brighter environment because fewer trees create structure for shadows and should therefore lead to a higher albedo.

Agriculture is an active area of development in CLM [12–81]. Here, we show that, for the recent past, crops are mostly well represented in this region as a mix of corn and soybeans. The next most common crop, alfalfa (a perennial legume), is not represented at all by CLM, and in these grid cells CLM classifies the rest of the crop area as spring wheat, which is almost never grown in this region, but serves to more generically represent cereal crops that are not parameterized. As new parameterizations are developed for more crops, the CLM crop model will diversify (see table 26.1 in [74]).

It is noteworthy that CLM assumes a relatively stable crop fraction over the past 70 years, while county records show a substantial drop in crop cover from >70% in the 1950s. While there is some debate about the significance of land abandonment in the U.S. [82], this region of Michigan has a clear, substantial drop that is not represented in CLM. The timing of this drop coincides with the expansion of suburban areas in Michigan and the rest of the U.S. Upper Midwest [83]. That CLM does not capture these large changes in land use over the recent past suggests that the model should not be used to assess environmental impacts of urbanization at this scale and in this setting. Historical patterns of irrigation are well represented, however. Irrigation in the area began in the early 1950s, as indicated by NASS data, and has slowly increased since then.

### 4.2. Model/Data Outputs (Dis)Agreement

LAI, productivity, soil moisture, NDVI, and albedo are all predicted values in CLM that can be compared to measured or satellite-estimated values. Of these, LAI performed the worst in comparison to satellite estimated LAI. In CLM plants begin to grow later in the season, increase to unrealistically high values in the summer, then hold on to their leaves well past the end of the growing season, sometimes into January of the following year. The importance of phenology as an indicator of the carbon cycle and plant productivity has been emphasized in the LSM literature (e.g., [84]); however, that does not appear to be the case here.

Productivity estimates from the model (GPP and NEE, Figure 7C,D) match much more closely both in mean values and the seasonal cycle. The EC tower measurements demonstrate the large amount of variability among landscape patches, even though these seven towers were in relatively similar landscapes (agriculture and restored prairie). Since the CLM PFTs are mostly agriculture and grass, this comparison likely works better than it would if CLM accurately represented the land cover in this area. The CLM shows little interannual variability; however, the EC towers detect substantial differences from year to year. From these data, only located in herbaceous areas, it is impossible to assess whether this interannual variability is due to grid cell scale variation in productivity. It is possible that this variability would be dampened if EC tower data were available from a wider range of land cover types, like forests, that might be more stable under varying climatic conditions.

Soil moisture during the growing season (gray areas in Figure 8) showed consistent patterns between CLM and SMAP, though a substantial difference in magnitude. Though only three years of data were available, the temporal patterns of soil moisture in CLM follow SMAP well. However, summertime soils in CLM are wetter than is observed by SMAP which is surprising given that CLM meteorology is fairly accurate, and the high LAI in CLM would suggest more transpiration. It is possible, however, that the shallower rooting depth prescribed for grasses in CLM [74] could lead to less transpiration and, therefore, higher soil moisture. Soil and plant hydraulics are active areas of research in CLM [11–25], and our analysis suggests that, at this site, the within summer seasonal patterns are nearly correct but the overall magnitude could still be improved.

Surface reflectance measures are important to consider for two reasons. First, albedo is a key driver of surface temperatures and so essential when ESMs are used to predict climate change [85]. Second, satellites measure incoming and reflected radiation, and so from a benchmarking perspective these are some of the least modified measures of land surface properties. With NDVI, there is a spectral mismatch between MODIS NDVI and NDVI calculated from CLM outputs, since the visible reflectance in CLM is calculated across the whole visible spectrum, and in eMODIS NDVI only the red band (620–670 nm) is used [86]. We would expect this mismatch to lead to a dampened NDVI in CLM, because the difference between the NIR and the entire visible spectrum should be less than the difference between the NIR and the red band alone. We do observe this mismatch, with eMODIS NDVI annual mean and maximum values being significantly higher than CLM values. In comparison to the LAI mismatches, the NDVI mismatch is considerably smaller, however, which is likely because LAI values in this area are in the range where the NDVI/LAI relationship begins to saturate (~4; [87]), and so the very high LAI values in CLM would not be captured by NDVI. The low values of NDVI in the winter reflect the higher snow cover in CLM, which are shown in the individual albedo streams.

We show that CLM albedo estimates were much different from MODIS in the summer, with the annual mean and maximum values all significantly different, and some of the NIR time series having negative correlations (Table S4). In contrast, while the winter CLM albedo values were still, on average, significantly different from MODIS, the time series performed reasonably well, with all the time series correlation tests having significant relationships. Average maximum albedo values in CLM were too high and corroborates what we showed with NDVI—that CLM snow is more prevalent than is observed in MODIS albedo products.

One year that is particularly interesting is 2012, a substantial drought year across the Midwest [76]. The effects of the drought are evident in the EC tower data (Figure 7C,D), in winter MODIS NDVI and albedo (Figure 7E–H), and low precipitation in 2012 is evident in the precipitation data both in the weather station and CRU-JRA data streams (Figure 4C). The drought impacts on LAI, however, are barely detectable both in the CLM and in the MODIS LAI product, though the seasonality of LAI is somewhat different in this year as compared to other years (Figure 7A). In the case of CLM LAI, this lack of a drought signal is because LAI is determined by the leaf carbon pool stored in the previous year; plants must spend their leaf carbon pool on leaves in the upcoming growing season regardless of current environmental conditions [74]. CLM grass PFTs are drought deciduous and so may respond to low soil moisture and lose leaves [23], though given the higher soil moisture in CLM and the relatively high threshold to trigger this effect, it is unlikely to get triggered here. Temperate broadleaf deciduous trees in CLM do not lose leaves in response to drought, though they may limit photosynthesis if water is unavailable [74]. The MODIS LAI product also does not respond to drought in a substantial way, though the time series (Figure 7A) does show a different shape and shorter time at peak greenness in 2012. As the MODIS LAI algorithm is based on a look up table that relies on red and NIR reflectance [65] we expect that this mismatch is again due to saturation of the relationship between NDVI and LAI at LAI values of around 4.

In all, it is reassuring that different ways of thinking about productivity and reflectance in CLM do not seem to compound to create larger uncertainties. Here, we show that LAI in CLM is incorrect in both magnitude and timing, yet this does not seem to impact the timing or magnitude of productivity. In CLM, senescence is delayed much longer than is realistic. This delay is likely due to the unrealistic dominance of grasses in these grid cells, which require very cold temperatures or very short days to senesce. Our productivity comparisons suggest, however, that physiological parameters still must be accurate enough such that productivity is shutting down at the right time seasonally. NDVI values match reasonably well given mismatches in the ways they are calculated, and albedo values follow our expectations given the land cover mismatches between CLM and observations. The pattern of soil moisture in CLM is comparable to that measured by SMAP; however, the soil in CLM is much wetter.

### 4.3. Future Work

Despite CLM’s poor performance at the local landscape scale in many of the metrics compared here, other studies have shown that, when CLM is run with local conditions and meteorological forcing, it can perform well [88]. Future work in this watershed will include running CLM at the local scale using observed changes in land cover, land use practices, and meteorological forcing. Running the model with more appropriate forcing data will allow us to both better understand the carbon/water cycle in this watershed and better understand which components of the CLM are capturing real variability and which are missing important processes. While at a monthly and annual time step, we showed that the meteorological forcing data matched the station data fairly well, it is possible that capturing finer temporal scale variation in weather patterns would improve model performance. We have also established a new network of EC towers in the KW that include more variety of PFTs, including forest, wetland, and urban sites [89]. These new data (2018 was the first full year of collection) will allow us to better understand carbon dynamics in this region.

Another area where more research would dramatically improve the model is in land cover and PFT mapping. Weaknesses in land cover maps have been identified by other researchers as well [90]. Here, we note that the early-to-mid 20th century is an era where data exist for much of the world (aerial and ground photo archives, historical records), but these data are dispersed and time consuming to access and analyze. An effort to digitize, process, and share these data would substantially improve our ability to simulate land surface processes in the recent past.

For comparisons with satellite imagery, one area where CLM evaluation methods could be improved is in radiative transfer modeling (RTM). In CLM, light is partitioned into direct and diffuse visible and NIR bins, but specific red, blue, NIR, and SWIR wavelength ranges could be modeled which would make comparisons between CLM and passive optical satellites more direct.

## 5. Conclusions

LSMs help us understand how land patterns and processes change in space and time at coarse spatial resolutions and large spatial extents. They are used to both understand past and present carbon, water, and energy cycling, and to predict future conditions. However, global models can be inaccurate when considered at more heterogeneous local scales. We took a cross-disciplinary approach to model evaluation, considering meteorology, land cover mapping, plant physiology, hydrology, and reflectance at the landscape scale. We showed that, on the input side, meteorological forcing data matches observations reasonably well, but land cover/PFT mapping and snow cover are key sources of uncertainty in CLM. Ecohydrology is an additional area of concern in the model, as soils remain wetter than observations despite factors that would seemingly lead to drier soils, and drought responses in CLM are not nearly as notable as they are in observations. Phenology could use more study, especially fall leaf drop, which, in CLM, lasts well into Michigan’s winter months. Surprisingly, the mismatch in phenology did not carry over to a mismatch in productivity, suggesting that while leaves remain on too long in CLM, they are not active, and they are not impacting the carbon cycle as much as might be expected. As LSMs advance in both complexity and spatial resolution, it is essential that we consider how the patterns and processes that are represented affect one another. This work highlights the importance of considering model uncertainty when LSMs or ESMs are used to inform land use decision making.

## Supplementary Material

Supplementary material
