# Supplementary material for: Challenging a Global Land Surface Model in a Local Socio-Environmental System
Source: Land (Basel). Author manuscript; Available in PMC 2021 Mar 8. (PMC7939037; doi:10.3390/land9100398)
Supplement: Supplementary material [file NIHMS1671081-supplement-Supplementary_material.docx]

Supplementary Material for

**Challenging a global land surface model in a local socio-environmental system**

Dahlin KM^1,2^, Akanga D^1^, Lombardozzi DL^3^, Reed DE^4^, Shirkey G^1,4^, Lei C^1,4^, Abraha M^4^, & J Chen^1,2,4^.

^1^Department of Geography, Environment, and Spatial Sciences. Michigan State University (MSU), East Lansing, Michigan, USA.

^2^Program in Ecology, Evolutionary Biology, and Behavior, MSU, East Lansing, Michigan, USA.

^3^National Center for Atmospheric Research, Boulder, Colorado, USA.

^4^Center for Global Change and Earth Observation, MSU, East Lansing, Michigan, USA.

* Corresponding Author email: kdahlin@msu.edu

**Table S1.** Land cover classes/plant functional types in CLM, NLCD, CropScape, and our Landsat classifications identified in the KW, CLM-N, and/or CLM-S.

| CLM5.0 land unit | CLM5.0 plant / crop functional types | NLCD (2001 - 2016) | CropScape (2008-2018) | Landsat Classification (1976 - 2015) |
| --- | --- | --- | --- | --- |
| **Vegetated** (“natural”) | Temperate needle leaf evergreen trees (NET) | Evergreen Forest | Evergreen Forest | Forest |
|  | Temperate broad leaf deciduous trees (BDT) | Deciduous Forest | Deciduous Forest | Forest |
|  | BDT* | Mixed Forest | Mixed Forest | Forest |
|  | NR | Shrub/Scrub | Shrubland | NR |
|  | C3 grasses (C3) | Grassland/  Herbaceous | Grass/ Pasture | Grass |
|  | C4 grasses (C4) | Pasture / Hay | Grass/ Pasture | Grass |
|  | Bare ground | Barren Land | Barren | Barren |
|  | BDT** | Woody Wetlands | Woody wetlands | Wetland |
|  | C3** | Emergent herbaceous wetlands | Herbaceous Wetlands | Wetland |
| **Crop** | Temperate corn | Cultivated Crops | Corn | Crop |
|  | Temperate irrigated corn | Cultivated Crops | Corn**** | Crop |
|  | Spring wheat | Cultivated Crops | Spring wheat | Crop |
|  | Irrigated spring wheat | Cultivated Crops | Spring wheat**** | Crop |
|  | Temperate Soybean | Cultivated Crops | Soybeans | Crop |
|  | Temperate irrigated soybean | Cultivated Crops | Soybeans**** | Crop |
|  | NR | Cultivated Crops | Alfalfa | Crop |
|  | NR | Cultivated Crops | Winter Wheat | Crop |
|  | NR | Cultivated Crops | Idle/ Fallow cropland | Grass |
|  | NR | Cultivated Crops | >40 other crops | Crop |
| **Urban** | High Density (HD) | Developed, high intensity | Developed/ High Intensity | Urban |
|  | Medium Density (MD) | Developed, Medium Intensity | Developed/ Medium Intensity | Urban |
|  | Tall Building District (TBD) | Developed, high intensity | Developed/ High Intensity | Urban (maybe NA) |
|  | C3 | Developed, Low intensity | Developed/ Low Intensity | Urban |
|  | C3 | Developed, open space | Developed/ Open Space | NR |
| **Lake** | *** | Open Water | Open water | Water |

NR = not represented in study region during time period of interest.

* = Mixed forests are classified as BDT because this is the more common tree type. Mixed forests make up a small fraction of NLCD cover in this region (<1%).

** = Wetlands in CLM are represented dynamically as inundated land.

*** = Small, shallow lakes are not represented in CLM. Only large, deep lakes are represented, which are not present in CLM-N or CLM-S.

**** = Irrigated area estimated based on county-level data from USDA NASS.

**Table S2.** Cross-walking table for presettlement vegetation cover classes with coarser vegetation classes for comparison with CLM and other maps.

| **Cover Class** | **Coarse Class** | **Detailed Class** |
| --- | --- | --- |
| Alder/Willow Swamp | forest | deciduous forest |
| Aspen Swamp | forest | deciduous forest |
| B Oak/W Oak | forest | deciduous forest |
| Beech/S Maple | forest | deciduous forest |
| Beech/S Maple/B Wood/R Oak | forest | deciduous forest |
| Black Ash Swamp | forest | deciduous forest |
| Black Spruce Swamp | forest | evergreen forest |
| Black Willow Swamp | forest | deciduous forest |
| Bog | wetland | wetland |
| Bur Oak Savanna | grass | grass |
| Cedar Swamp | forest | evergreen forest |
| Conifer/Hardwood Swamp | forest | evergreen forest |
| Dogwood/Willow Swamp | forest | deciduous forest |
| Elm Swamp | forest | deciduous forest |
| Emergent Marsh | wetland | wetland |
| Hardwood/Conifer Swamp | forest | deciduous forest |
| Hemlock/W Pine | forest | evergreen forest |
| Inland Salt Marsh | wetland | wetland |
| Intermit Wetland | wetland | wetland |
| Lake | water | water |
| Low Conifer Swamp | forest | evergreen forest |
| Low Hardwood Swamp | forest | deciduous forest |
| Oak Barrens | forest | deciduous forest |
| Oak Opening | grass | grass |
| Oak/Pine Barrens | forest | deciduous forest |
| Outcrop | barren | barren |
| Pine Barrens | forest | evergreen forest |
| River | water | water |
| Silver/Red Maple Swamp | forest | deciduous forest |
| Tamarack Swamp | forest | deciduous forest |
| Upland Grassland | grass | grass |
| W Oak/Hickory | forest | deciduous forest |
| W Pine/Beech/R Maple | forest | evergreen forest |
| W Pine/W Oak | forest | evergreen forest |
| Wet Meadow | grass | grass |
| Wet Prairie | grass | grass |
| White Pine | forest | evergreen forest |
| White Pine Swamp | forest | evergreen forest |
| Alder/Willow Swamp | forest | deciduous forest |
| B Oak/W Oak | forest | deciduous forest |
| Beech/S Maple/B Wood/R Oak | forest | deciduous forest |
| Black Ash Swamp | forest | deciduous forest |
| Black Willow Swamp | forest | deciduous forest |
| Dogwood/Willow Swamp | forest | deciduous forest |
| Elm Swamp | forest | deciduous forest |
| Hardwood/Conifer Swamp | forest | deciduous forest |
| Low Hardwood Swamp | forest | deciduous forest |
| Oak Barrens | forest | deciduous forest |
| Silver/Red Maple Swamp | forest | deciduous forest |
| Tamarack Swamp | forest | deciduous forest |
| W Oak/Hickory | forest | deciduous forest |
| Balsam Poplar Swamp | forest | deciduous forest |
| Beach | barren | barren |
| Beech/Hemlock | forest | deciduous forest |
| Cottonwood Swamp | forest | deciduous forest |
| Hemlock | forest | evergreen forest |
| Hemlock Swamp | forest | evergreen forest |
| Lake Plain Prairie | grass | grass |
| Open Sand Dune | barren | barren |

**Table S3.** Percent land cover or functional type for CLM versus NLCD and CropScape (CS) in 2016.

| **LC/PFT** | **CLM-N** | **CLM-S** | **NLCD/ CS-N** | **NLDC/ CS-S** | **NLCD/ CS-KW** | **Mean Difference*** | **Normalized Difference**** |
| --- | --- | --- | --- | --- | --- | --- | --- |
| Bare Ground | 0 | 0 | 0.16 | 0.12 | 0.25 | -0.18 | -1.00 |
| BDT Forest | 10.21 | 7.81 | 29.73 | 27.29 | 36.28 | -22.1 | -0.55 |
| NET Forest | 1.00 | 0.73 | 0.60 | 0.31 | 0.68 | 0.34 | 0.24 |
| C3 Grass | 35.97 | 33.15 | 10.75 | 9.91 | 12.54 | 23.49 | 0.51 |
| C4 Grass | 4.99 | 9.45 | 5.46 | 6.76 | 5.17 | 1.42 | 0.11 |
| HD Urban | 0.014 | 0.0059 | 0.61 | 0.51 | 0.67 | -0.59 | -0.97 |
| MD Urban | 0.43 | 0.23 | 1.45 | 1.06 | 1.52 | -1.01 | -0.61 |
| Corn | 18.36 | 21.64 | 17.31 | 21.78 | 16.58 | 1.44 | 0.037 |
| Soybeans | 18.68 | 22.04 | 15.41 | 18.56 | 11.10 | 5.33 | 0.15 |
| Other crops | 10.3 | 4.96 | 12.93 | 6.72 | 8.13 | -1.63 | -0.097 |

* = Mean difference refers to the difference of the mean of CLM values minus the mean of the NLCD/CS values.

** = Normalized Difference = (mean CLM – mean NLCD) / (mean CLM + mean NLCD)

**Table S4.** Comparison of CLM and MODIS mean and maximum albedo values for black sky (direct) and white sky (diffuse) in the visible and NIR wavelength ranges. WS = white sky, BS = black sky, DIF = diffuse, DIR = direct, MOD =MODIS, max = comparison of maximum values, mean = comparison of mean values, R = Pearson correlation coefficient, *ρ* = Spearman’s rank correlation, ns = not significant.

|  | CLM mean | CLM max | MOD mean | MOD max | Max t-test | Mean t-test | Max R | Max *ρ* | Mean R | Mean *ρ* |
| --- | --- | --- | --- | --- | --- | --- | --- | --- | --- | --- |
| Summer | | | | | | | | | | |
| BS/DIR NIR | 0.22 | 0.25 | 0.25 | 0.27 | p < 0.001 | p < 0.001 | ns | ns | -0.72 p = 0.002 | -0.63, p = 0.01 |
| BS/DIR visible | 0.054 | 0.084 | 0.042 | 0.055 | p < 0.001 | p < 0.001 | ns | ns | ns | ns |
| WS/DIF NIR | 0.29 | 0.29 | 0.27 | 0.30 | p = 0.02 | p < 0.001 | -0.68, p = 0.005 | -0.54, p = 0.04 | -0.68, p = 0.005 | -0.55, p = 0.04 |
| WS/DIF visible | 0.073 | 0.095 | 0.046 | 0.056 | p < 0.001 | p < 0.001 | ns | ns | ns | ns |
| Winter | | | | | | | | | | |
| BS/DIR NIR | 0.36 | 0.48 | 0.31 | 0.45 | ns | P < 0.001 | 0.69, p = 0.004 | 0.61, p = 0.02 | 0.61, p = 0.02 | 0.49, p = 0.06 |
| BS/DIR visible | 0.48 | 0.75 | 0.29 | 0.55 | p < 0.001 | p < 0.001 | 0.69, p = 0.004 | 0.53, p = 0.05 | 0.64, p = 0.01 | 0.57, p = 0.03 |
| WS/DIF NIR | 0.41 | 0.54 | 0.31 | 0.44 | p < 0.001 | P < 0.001 | 0.72, p = 0.002 | 0.72, p = 0.004 | 0.62, p = 0.01 | 0.54, p = 0.04 |
| WS/DIF visible | 0.48 | 0.74 | 0.28 | 0.54 | p < 0.001 | p < 0.001 | 0.70, p = 0.004 | 0.54, p = 0.04 | 0.64, p = 0.01 | 0.53, 0.05 |
